# A novel synthetic approach of cerium oxide nanoparticles with improved biomedical activity

**DOI:** 10.1038/s41598-017-04098-6

**Published:** 2017-07-05

**Authors:** Fanny Caputo, Marta Mameli, Andrzej Sienkiewicz, Silvia Licoccia, Francesco Stellacci, Lina Ghibelli, Enrico Traversa

**Affiliations:** 1 Dipartimento di Scienze e Tecnologie Chimiche, Università di Roma Tor Vergata, 00133 Roma, Italy; 2 Dipartimento di Biologia, Università di Roma Tor Vergata, 00133 Roma, Italy; 3 Institute of Materials, École Polytechnique Fédérale de Lausanne, 1015 Lausanne, Switzerland; 4 Institute of Physics, École Polytechnique Fédérale de Lausanne, 1015 Lausanne, Switzerland; 5 International Research Center for Renewable Energy, Xi’an Jiaotong University, 710049 Xi’an, Shaanxi China

## Abstract

Cerium oxide nanoparticles (CNPs) are novel synthetic antioxidant agents proposed for treating oxidative stress-related diseases. The synthesis of high-quality CNPs for biomedical applications remains a challenging task. A major concern for a safe use of CNPs as pharmacological agents is their tendency to agglomerate. Herein we present a simple direct precipitation approach, exploiting ethylene glycol as synthesis co-factor, to synthesize at room temperature nanocrystalline sub-10 nm CNPs, followed by a surface silanization approach to improve nanoparticle dispersibility in biological fluids. CNPs were characterized using transmission electron microscopy (TEM) observations, X-ray diffraction (XRD) analysis, thermogravimetric analysis (TGA), Fourier-transform infrared (FT-IR) spectroscopy, proton nuclear magnetic resonance (^1^H-NMR) spectroscopy, dynamic light scattering (DLS) and zeta potential measurements. CNP redox activity was studied in abiotic systems using electron spin resonance (ESR) measurements, and *in vitro* on human cell models. *In-situ* silanization improved CNP colloidal stability, in comparison with non-functionalized particles, and allowed at the same time improving their original biological activity, yielding thus functionalized CNPs suitable for biomedical applications.

## Introduction

Cerium oxide (CeO_2_) is a rare earth oxide material used in many technological applications^1^. These include a wide range of catalytic applications^2^, oxygen sensors^3^, oxygen permeation membranes^4^, solid oxide fuel cells^5–7^, glass-polishing^8^, and as an ultraviolet absorbent^9–12^. In the last decade cerium oxide nanoparticles (CNPs) have gained a significant interest in the medical field, thanks to their self regenerating antioxidant properties, representing a promising antioxidant for healing numerous untreatable oxidative-stress-related diseases, as discussed in several reviews^13–18^. CNPs may help to solve molecular antioxidant drugs limitations, such as difficulties in body retention, stability and selectivity^19, 20^, significantly improving the usefulness of antioxidant therapy.

The Ce^3+^/Ce^4+^ redox couple on the nanoparticle surface provides CNP biological activity. Their action mechanism is similar to that of natural metallo-enzymes that use transition metal ions, such as Fe^3+^, Cu^2+^, or Mn^3+^, to buffer reactive oxygen species (ROS) in cells and tissues. Indeed, Ce^3+^ ions reduce superoxide into hydrogen peroxide while oxidizing into Ce^4+^, mimicking thus superoxide dismutase (SOD) activity^21, 22^. Also, oxidation of Ce^3+^ to Ce^4+^ permits abating other deleterious free radicals, such as hydroxyl^23^, nitric oxide (NO)^24^, and peroxynitrite (ONOO−)^25^. Instead, the reduction of Ce^4+^ to Ce^3+^ induces hydrogen peroxide oxidation to molecular oxygen, behaving like catalase enzyme^26^.

Owing to their antiradical activity, different studies have shown that CNPs help reducing symptoms of many oxidative stress-related diseases, including neuro-degenerations^27, 28^, retinitis^29^, chronic inflammation^30^, diabetes^31^, endometriosis^32^, and cancer^33^. Therefore, the demand of CNPs into the medical field is rapidly expanding. However, the synthesis of high quality CNPs suitable for biomedical applications is still a challenging task^34, 35^.

One of the major issues that still needs to be solved for a safe and efficient use of CNPs as pharmacological agents is their tendency to agglomerate, experienced when CNPs are suspended in aqueous solutions as well as *in vivo* and in physiological media^15^. The formation of precipitates is expected to decrease the active surface area of CNPs, thereby reducing their biological activity^36^. Importantly, nanoparticle agglomeration could cause toxicity and deleterious side effects^37, 38^. If administered *in vivo*, nanoparticle agglomerates may accumulate in target organs, such as spleen and kidney^39^, possibly causing the failure of their functions. Moreover, it has been recently demonstrated that big nanoparticle agglomerates induce incomplete phagocytosis phenomena in macrophages depressing the inflammatory response and producing cytotoxic cytokines^40^. Therefore, it is becoming very important to develop robust synthesis methods to obtain water-dispersible CNPs.

Organic solvent-assisted synthesis^41^ and micro-emulsion approaches^42, 43^ have been proposed to synthesize stable CNP colloidal dispersions. However, in the above-mentioned methods not totally biocompatible surfactants or small organic molecules (e.g. oleic acid) are commonly used to transfer CNPs to water based media, causing toxic effects on cells^15, 41^.

Low temperature direct precipitation of nanocrystalline CNPs in water^7, 34^ has been also proposed as a synthetic way to obtain biocompatible CNPs for medical applications^44–46^. The use of biocompatible polymers such as PEG and dextran has been explored to control the reaction kinetics^15, 44, 47^. After the synthesis, a residual polymeric layer remains on the CNPs^44, 48^, which should be permeable to free radicals, and should not interfere with CPN redox chemistry to avoid the inhibition of the CNP antioxidant activity. To enhance the CNP stability and to improve selectivity in CNP-cell interactions, post synthesis modification strategies of the CNP ligand shell has also been explored^43, 49^.

In this work, we present a suitable approach to produce biocompatible and redox active CNPs for biomedical use. For this purpose, we optimized two direct-precipitation synthetic ways, in particular exploiting ethylene glycol as synthesis co-factor, for synthesizing at room temperature nanocrystalline sub-10 nm CNPs, and then combined with a new post-synthesis surface functionalization strategy to improve nanoparticle dispersibility in biological fluids. CNP agglomeration in water-based media, redox activity, bio-compatibility and antioxidant efficiency in a human lymphocyte cellular model were assessed with the aim to select the most suitable approach to obtain CNPs for biomedical applications.

## Results and Discussion

### CNP synthesis and physico-chemical properties

Five different CNP samples were synthesized using two different precipitation methods, as summarized in Table 1.

**Table 1:** CNP samples prepared with method 1: TEMED induced precipitation in the presence of Pluronic F127; and method 2: NH_3_ induced precipitation in the presence of EG.

Sample	Synthetic method	Surface functionalities
C1	1	Pluronic F127
C1–450 °C	1	None (annealed at 450 °C)
C2	2	Ethylene glycol
C3	2	MEEETES
C4	2	APTES

TEMED: N,N,N0,N0-tetramethylethylenediamine; EG: ethylene glycol; APTES: (3-aminopropyl)triethoxysilane; MEEETES: 6-{2-[2-(2-Methoxy-ethoxy)-ethoxy]-ethoxy}-hexyl)triethoxysilane.

#### TEMED-induced precipitation in the presence of Pluronic F127

As a first attempt, the N,N,N0,N0-tetramethylethylenediamine (TEMED)-induced precipitation reported by Esposito and Traversa^7^ was slightly modified. This synthetic strategy is based on the use of a biocompatible polymer, Pluronic F127, as a synthesis co-factor to produce high quality crystalline CNPs^7^. The nanoparticles obtained have been previously tested in several cellular models, showing a good antioxidant activity^36, 45, 46, 50^. However, in these works the CNPs, which were already crystalline after precipitation, were subjected to a post-synthesis thermal treatment to completely eliminate the organic residual layer on the nanoparticle surface, inducing a strong tendency to form agglomerates that limit their use *in vivo* ^36^. Therefore, here the post-synthesis thermal treatment of the CNPs was substituted by a new washing procedure to selectively eliminate the toxic precipitating agent (TEMED), maintaining the polymeric superficial coating (method 1).

**Figure 1:**
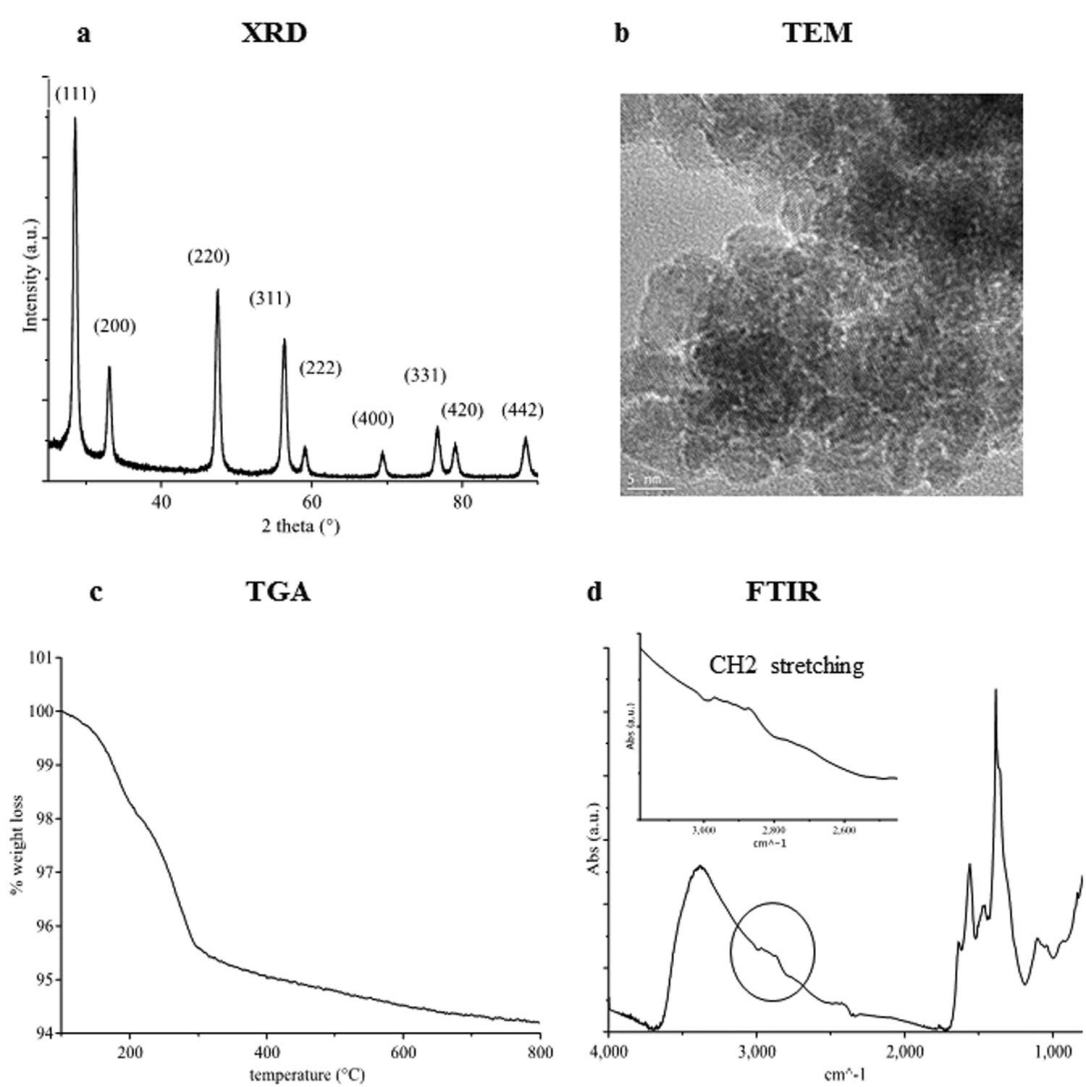
Physico-chemical characterization of C1 nanoparticles. (**a**) XRD diffraction patterns and Miller indexes of C1. (**b**) TEM image. (**c**) TGA curve. (**d**) FTIR absorbance spectra with the inset of the CH_2_ stretching zone.

Figure 1a shows the XRD pattern and Miller indexes for the as-dried powders obtained with this method (named C1, see Table 1), having the crystalline features of the fluorite structure. Scherrer analysis on the (111) peak showed an average crystallite size of 8 nm. TEM observations revealed that the powder consisted in nanometric semispherical and hexagonal particles with grain size in the 4–10 nm range (Fig. 1b). TGA measurements (Fig. 1c) showed a first weight loss of about 1.5 wt% below 200 °C, which was associated with the volatilization of the residual solvent and adsorbed water. Then the sample presented a weight loss of about 4 wt% between 200 and 700 °C, which was attributed to the decomposition of the organic coating on the CNP surface. The nature of this coating was studied by FTIR (Fig. 1d) and ^1^H-NMR (Supplementary Fig. S1) measurements. No traces of free TEMED were detected by the ^1^H-NMR analysis, indicating that the toxic amine compound was successfully removed by the washing procedure.

FTIR and ^1^H-NMR analysis showed the presence of Pluronic F127. FTIR spectra showed different peaks between 2970 and 2870 cm^−1^ belonging to methyl, methylene and methine stretching. The ^1^H-NMR showed three peaks typical of Pluronic F127 at 1.15, 3.56, and 3.75 ppm, which are associated with the hydrogen ions of the free methyl of the CHO-CH_3_ group, of the methine of the CH-O-H group, and of the methylene in the CH_2_-CH_2_-O and O-CH_2_-CH_2_-O groups, respectively^51^. The signals associated to Pluronic F127 shown in the IR spectrum were not very pronounced, in agreement with the low mass loss detected by TGA measurements, possibly indicating a limited coordination of the polymer on the CNP surface. The small amount of absorbed Pluronic F127 is not sufficient to significantly improve CNP water dispersibility.

Thermally treated CNPs, named C1–450 (see Table 1), were produced for sake of comparison with the previous work of our group^36, 45^. Supplementary Figure S2 shows the physico-chemical characterization of the C1–450 samples. As expected, the thermal treatment at 450 °C completely removed the organic layer without incrementing the average grain size^7^.

#### Ethylene glycol-assisted precipitation

Another synthesis approach was adopted (method 2 in Table 1) to avoid the occurrence of a polymeric layer on the CNP surface and to improve CNP water dispersibility. We took advantage of the ethylene glycol (EG) ability to complex Ce^3+^ ions to control the reaction homogeneity, setting up a simple ammonia induced direct precipitation process. Similar low temperature direct precipitation synthesis of CNPs in EG/water mixed solvents was previously described by other groups^34, 52^. Herein the reaction conditions reported in ref. 52 were optimized by modifying the ethylene glycol concentration and by choosing the pH to maximize EG coordination to Ce^3+^. EG to Ce^3+^ molar ratio was chosen to be 10 to 1, slightly larger than the theoretical Ce^3+^ coordination ratio in the presence of nitrate ions^53^. The coordination dependence upon the variation of the pH was studied by ^1^H-NMR titration (Supplementary Fig. S3), analyzing the EG peak broadening at 3.66 ppm. Paramagnetic lanthanide ions such as Ce^3+^ are known in NMR measurements for their ability to induce paramagnetic relaxation enhancement (PRE), an effect that causes the signals of the resonant spins surrounding the metal ion to broaden once they are coordinated to the metal^54^. On this basis, to study the Ce^3+^-ethylene glycol binding, the pH was varied between 3 and 11. As shown in Supplementary Fig. S3, a maximum in EG peak broadening was reached between 9.2 and 9.7; hence the reaction pH was fixed at 9.6.

**Figure 2:**
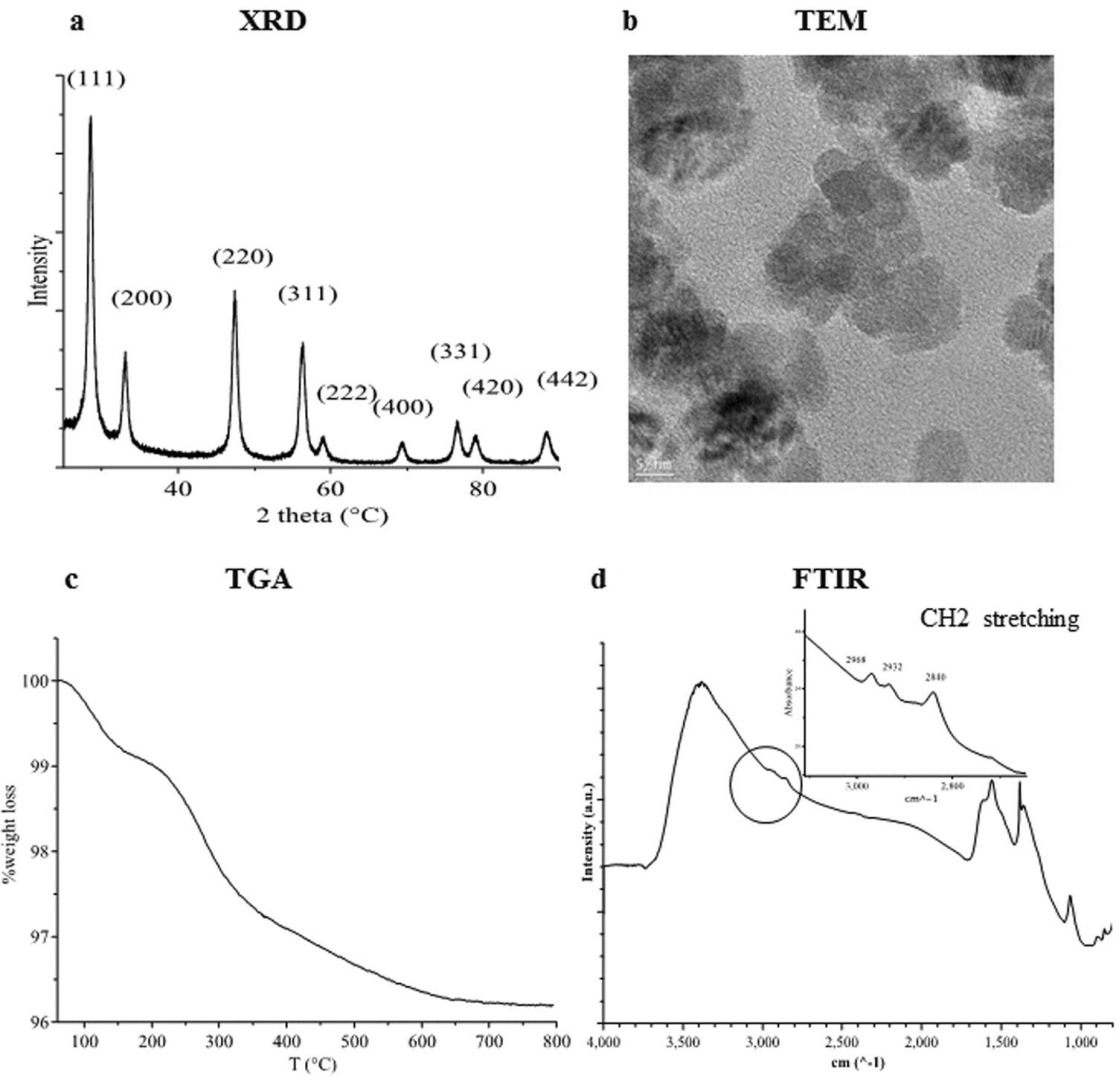
Physico-chemical characterization of C2 nanoparticles. (**a**) XRD diffraction patterns and Miller indexes. (**b**) TEM image. (**c**) TGA curve. (**d**) FTIR absorbance spectra; the inset shows the CH_2_ stretching zone.

Figure 2 shows the physico-chemical characterization of the obtained CNPs (C2, see Table 1). As shown in Fig. 2a, C2 particles were already crystalline after drying and showed a fluorite structure, with an average dimension (calculated by Scherrer analysis) of 11 nm. TEM analysis (Fig. 2b) showed mono-crystalline semispherical and hexagonal particles, with grain size ranging 7–15 nm. TGA analysis (Fig. 2c) showed two main regions of weight loss. The first loss of about 0.5 wt%, which occurred below 200 °C, was associated with the volatilization of the residual ethanol and water solvents, while the 2.7 wt% loss between 200 and 800 °C was associated with the decomposition of the ethylene glycol residual layer on the dried powder surface. The presence of a superficial EG coating was confirmed by the FTIR analysis (Fig. 2d), which showed three peaks between 2970 and 2840 cm^−1^ typical of the methylene stretching of ethylene glycol molecules^55^.

### CNP silanization

In order to obtain water dispersible CNPs, a post-synthesis functionalization strategy of C2 nanoparticles with small hydrophilic molecules was developed. Silanization of the CNPs was then performed taking advantage of the high reactivity of the silanes towards the hydroxyl groups present on the surface. The above-mentioned approach was largely employed by many groups to introduce PEG and amine functionalities onto magnetic oxide nanoparticles^56–58^, as well as for silica nanoparticles^59–61^, but was scarcely investigated for CNPs^62^. Two small hydrophilic biocompatible silane molecules, 6-{2-[2-(2-Methoxy-ethoxy)-ethoxy]-ethoxy}-hexyl)triethoxysilane (MEEETES) and 3-Aminopropyltriethoxysilane (APTES), were chosen for this purpose. APTES organo-silane was selected for the presence of the hydrophilic amino terminal group that can be used as a linker to allow further surface functionalization with a variety of bio-molecules. On the other hand MEEETES showed the best hydrophilic properties among all the organo-silane of our interest^63^.

**Figure 3:**
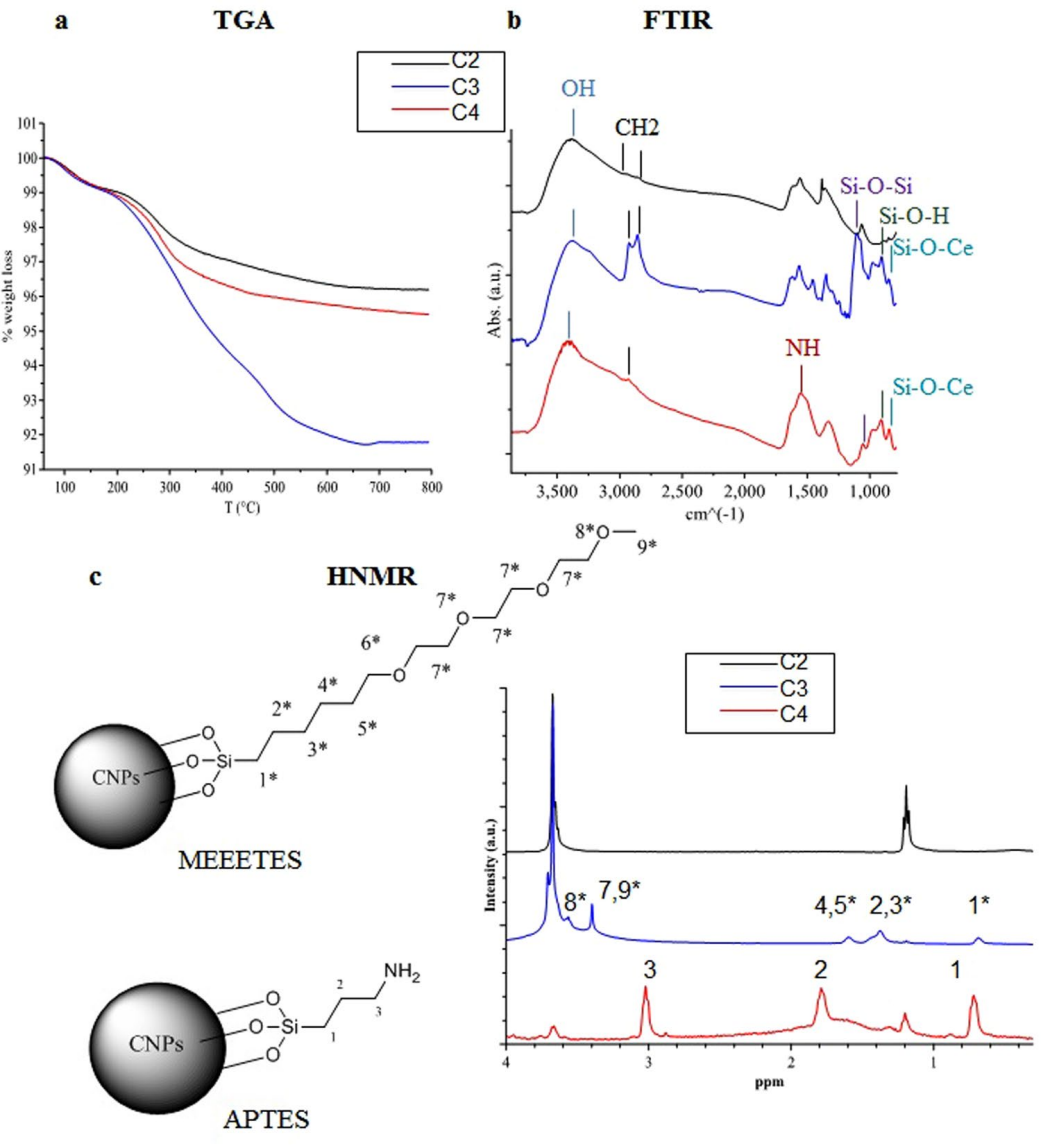
C2 nanoparticle surface silanization. (**a**) TGA analysis of C2, C3 and C4 samples. (**b**) FTIR absorbance spectra. (**c**) ^1^H-NMR spectra of C3 and C4 samples. The peaks were assigned to the APTES and MEEETES molecular structures.

Figure 3 shows the characterization of the samples after silanization. Figure 3a shows the TGA curves of the C3 (MEEETES) and C4 (APTES) samples compared with the C2 sample. Samples C3 and C4 showed a weight loss increment of 4.3% and 0.6% (*vs* C2 sample) between 200 and 700 °C, associated with the thermal decomposition of the silanes. These values are compatible with the formation of a silane monolayer on the CNP surface. FTIR (Fig. 3b) and ^1^H-NMR (Fig. 3c) analyses confirmed the presence of APTES and MEEETES ligand shell. The peaks between 2950 and 2850 cm^−1^ are due to CH_2_ stretching in the silane carbon chains. The introduction of silane ligands onto the CNP surface was demonstrated by the absorption bands in the 1130–1000 cm^−1^ and 950–810 cm^−1^ ranges, signals that are typical of siloxane and silanol groups, respectively^21, 22^. Importantly, the peak at 852 cm^−1^ due to the Ce-O-Si silanolate band indicated the formation of a covalent ligand binding between the silane ligands and the hydroxyl groups on the CNP surface^23, 64^. The presence of the NH bending band at 1554 cm^−1^, which can be ascribed to the amide bond, confirmed the effective functionalization of CNPs with APTES^24^. The ^1^H-NMR spectra (Fig. 3c) presented several broad peaks that can be assigned to the ligand molecules bound on the surface. Nine characteristic peaks for MEEETES and four for APTES were observed, which were attributed to the APTES and MEEETES chains, as shown in Fig. 3c, confirming the success of the functionalization strategy.

### CNP dispersion properties

CNP tendency to agglomerate in water and in physiological media was measured by DLS analysis. Table 2 summarizes the average hydrodynamic diameter of all the samples, while Supplementary Figure S4 shows the CNP size distribution profile reported by volume. With respect to C1, larger values of hydrodynamic diameter were observed for C1–450 in water, but only slightly larger in cell media. In addition, the C1 hydrodynamic diameter distribution presents two distinct populations of agglomerates, one at about 100 nm and the other at 250 nm (Fig. S4), being a highly polydispersed sample. It might be inferred that the presence of the Pluronic superficial coating only slightly reduced the nanoparticle tendency to agglomerate.

**Table 2:** Summary of DLS and zeta potential results for the tested CNPs. d: average hydrodynamic diameter ± standard deviation over three measurements obtained by cumulant analysis.

NPs	H_2_O		RPMI + 10% FBS	
	d (nm)	Z pot. (mV)	d (nm)	Z pot. (mV)
C1	164 ± 8	−23 ± 1	232 ± 9	−2 ± 1
C1–450	244 ± 5	−36 ± 1	276 ± 12	−7 ± 1
C2	222 ± 42	−27 ± 1	206 ± 7	−10 ± 1
C3	110 ± 8	−25 ± 1	110 ± 5	−10 ± 1
C4	1230 ± 60	14 ± 1	357 ± 23	−12 ± 1

Z_pot: zeta potential average ± standard deviation over three measurements.

**Figure 4:**
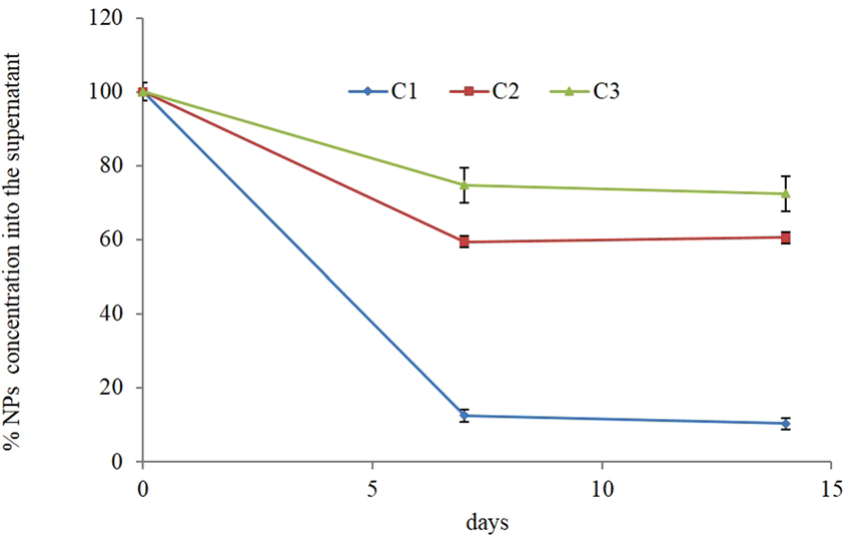
CNP colloidal stability over time. C1, C2, and C3 sample residual concentration into the supernatant after 7 and 14 days.

The dispersion stability of C1 nanoparticles in water (pH 7.4) was studied over time, following their precipitation for 14 days. C1 dispersion was let to sediment at ambient conditions; the CNP concentration into the supernatant was evaluated by measuring UV-Vis spectra, following the decrease of cerium absorbance peak around 300 nm^25, 34, 41^, as shown in Supplementary Fig. S5. C1–450 nanoparticles were excluded from the analysis since they completely precipitated within hours. Figure 4 shows that after 14 days only 10 wt% of the C1 nanoparticles was kept in the supernatant.

Therefore, by modifying the synthesis method reported by Esposito and Traversa^7^, the CNP dispersibility was partially improved, but the agglomeration issue was not satisfactorily solved. To obtain better results, the new synthetic and functionalization strategies were designed. DLS analysis of the CNPs obtained by EG assisted synthesis (C2) showed a lower tendency to agglomerate (*vs* C1). Their stability in water was studied over time, as described for the C1 sample (Fig. 4). C2 nanoparticles were much more stable in water, since more than 62 wt% of the original concentration remained stable into the supernatant for two weeks. However, the DLS hydrodynamic profile of C2 was characterized by a significant polydispersity (Supplementary Fig. S4).

As a next step, we explored the behavior of C2 after surface functionalization with APTES and MEEETES and we analyzed agglomeration and stability in water based media. CNPs functionalized with APTES (C4) presented a strong tendency to agglomerate. The hydrodynamic diameter distribution in water (Supplementary Fig. S4) showed a mono-dispersed population around 1 μm. Moreover, these NPs were completely precipitated within hours if left to sediment at room temperature. The low stability of the C4 formulation may be explained by crosslinking among CNPs during functionalization induced by the presence of the amine functionality in the APTES molecule, which facilitates the formation of siloxane bonds (detected by FT-IR, see Fig. 3b). Hence, our evidence clearly showed that CNP functionalization with APTES is not suitable for biomedical applications.

CNP functionalized with MEEETES (C3) were much less agglomerated than C2, presenting a population with an average hydrodynamic diameter peaked at 45 nm. Interestingly, C3 was the only sample that did not need a sonication procedure to be properly dispersed. Importantly, almost 80 wt% of the C3 nanoparticles remained into the supernatant after two weeks (Fig. 4), showing a very good stability in water-based media.

The surface charge of CNP samples was measured by zeta potential analysis, both in water (pH 7.4) and in cellular culture media. The average values are reported in Table 2. In water, CNP surface charge was clearly influenced by the surface coating, being negative in the case of Pluronic 127 (C1), MEEETES (C3), and in the presence of hydroxyl surface groups (C2 ad C1–450 °C), while it became positive in the presence of APTES, reflecting the presence of partially protonated APTES amino groups (C4). When CNP samples came in contact with serum proteins, the nanoparticles superficial charge was reduced to slightly negative values independently of the coating, meaning that the formation of a protein layer on the CNPs surface (protein corona) in physiological media completely shielded their original surface charge. The reduction of NP surface charge values induced more agglomeration phenomena in all the CNPs tested, except for the C3 sample: in this case, the mean hydrodynamic diameter of the main population was reduced from 45 to 30 nm.

### Radical scavenging by CNPs in abiotic systems

**Figure 5:**
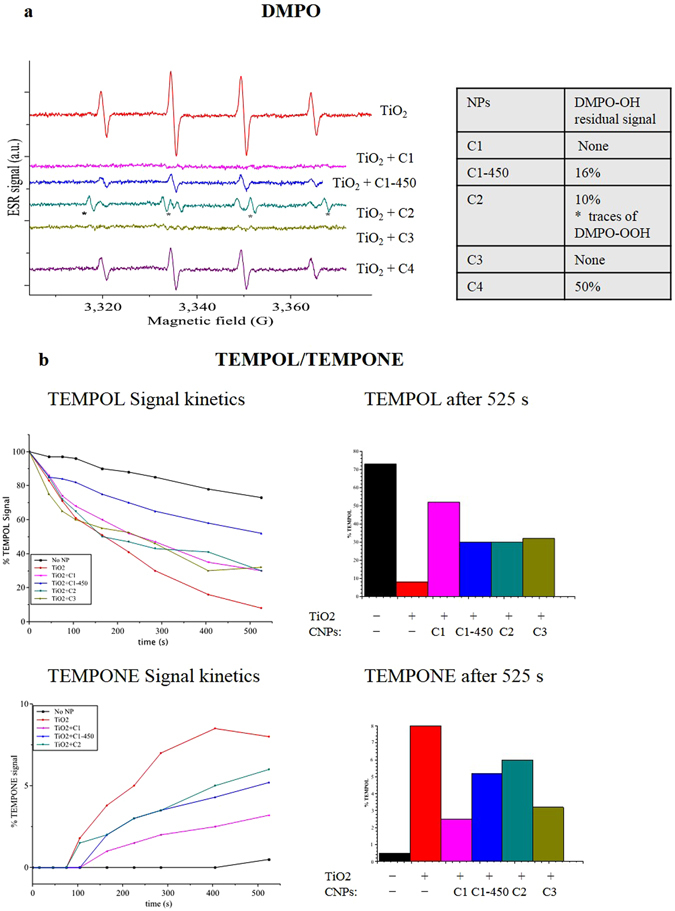
Abiotic analysis of CNP redox activity. (**a**) ESR spectra of DMPO spin abduct signals produced by UV-irradiated TiO_2_ suspensions at 800 µg/mL ± CNPs at 400 µg/mL. On the right a table reporting the DMPO-OH residual signal in the presence of C1, C1–450, C2, C3, and C4 samples. (**b**) ESR analysis of TEMPOL decomposition into TEMPONE during continuous irradiation of TiO_2_ (800 µg/mL) ± CNPs (400 µg/mL). On the left the TEMPOL signal reduction and the TEMPONE formation kinetics for 525 s, on the right % of TEMPOL disappearance and TEMPONE formation after 525 s.

The ability of CNPs to scavenge hydroxyl radical (HO^●^) was studied by electron spin resonance (ESR) measurements. Hydroxyl radicals were generated irradiating TiO_2_ nanoparticle dispersions with ultraviolet radiation, taking advantage of the well known TiO_2_ photocatalytic activity^65, 66^. Since the lifetime of HO^●^ at room temperature is very low (100 μs), the HO^●^ formation and decay in the presence of CNPs was studied using DMPO spin trap. The ESR spin trap technique takes advantage of spin trap molecule ability to react with free radicals, forming a more stable spin adduct with paramagnetic parameters that depend on the nature of the trapped radical^67^. In this work, the CNP redox activity was estimated by quantifying the formation of DMPO adducts in the absence (Fig. 5a, TiO_2_ spectrum) or in the presence of CNPs (Fig. 5a, TiO_2_ + CNPs spectra). In the absence of CNPs, the typical DMPO-OH spectrum was detected, consisting of four lines with peak intensity ratios of 1:2:2:1 and hyperfine coupling constants A_N_ and A_H_ for 14N and 1H equal to 14.9 G (Fig. 5a)^67, 68^. All the CNPs significantly reduced the DMPO-OH signal, showing a strong HO^●^ scavenging activity. The reduction of DMPO-HO signal was quantified by integrating the ESR spectra and reported in the table in Fig. 5a.

C1–450 and C2 reduced the DMPO-OH signal by 85–90%. C1 and C3 completely abolished the DMPO-OH signal. C4 scavenged HO^●^ much less than the other CNP samples, in agreement with their tendency to agglomerate; the reduced CNP active area impaired their redox activity. Interestingly, C2 spectra showed some traces of DMPO-OOH abduct characterized by a A_N_ 14.12 G, and A_H_ of 11.3 G hyperfine coupling constants^27, 67^, superimposed to the DMPO-OH residual spectra (Fig. 5a). Since no traces of superoxide forming DMPO-OOH were detected in the control spectra, this evidence indicates that C2 samples possess an additional activity, producing some extent of superoxide radicals while reacting with HO^●^.

The decomposition of 4-hydroxy-2,2,6,6-tetramethylpiperidine 1-oxyl (TEMPOL) free radical into 2,2,6,6-tetramethylpiperidinone 1-oxyl (TEMPONE) induced by HO^●^ was measured in the presence or in the absence of CNPs, as an alternative method to validate the reactivity of CNPs (Supplementary Fig. S6A). TEMPOL decomposition kinetics and the consequent TEMPONE formation were followed for 525 s, under a continuous generation of HO^●^. Figure 5b shows the increment of the TEMPONE signal over time; Supplementary Figure S6 shows the ESR spectra measured after 525 s. In agreement with the DMPO data, C1 and C3 nanoparticles were very active in scavenging HO^●^, reducing TEMPOL decomposition, and thereby TEMPONE formation by 70% and 62%, respectively. On the other hand, C2 and C1–450 only partially reduced the TEMPONE formation (35% and 25% reduction), being much less active.

Our findings indicate that CNP redox activity in abiotic systems is only partially correlated with their dispersibility. For example, C3 and C1 showed similar reactivity in scavenging HO^●^ free radicals but different dispersion properties (Supplementary Fig. S4). Moreover, C2 samples showed an interesting additional activity producing a small amount of superoxide. Therefore, we hypothesized that the CNP surface coating could play an important function in determining the CNP redox properties.

### CNP biocompatibility

**Figure 6:**
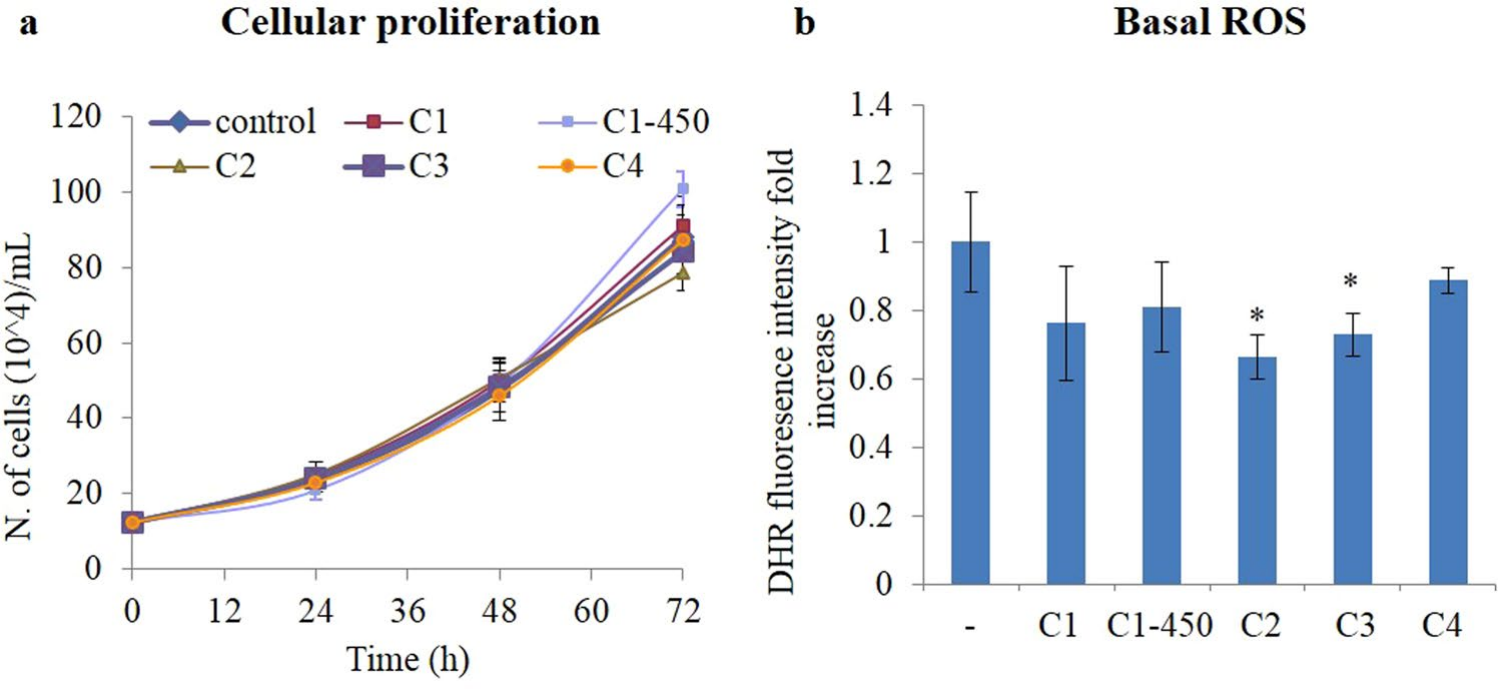
CNP nanoparticles do not show any toxicity. (**a**) Time course of cellular proliferation for 12–72 h measured by cells counting reported as number of cells. (**b**) Basal ROS levels measured by DHR fluorescent signal detected by flow cytometry 24 h after cell treatment with CNPs (200 μg/mL). Values are the mean of ≥3 independent experiments ± SD; *p < 0.05 (ANOVA). Significance with respect to the control group is shown.

It is well known that the CNP physico-chemical properties, including size^25, 28, 36^, shape^35, 69^, surface area^25, 30^, surface charge^31, 70^, and the presence of surface contaminants^32, 41, 44^, affect the CNP biocompatibility. Therefore, CNP effects on cell viability were tested in Jurkat human T lymphocytes, a mammal cell model previously systematically investigated in terms of cellular response to various exogenous stress sources^33, 36, 71^. Cells were treated with CNPs at a final concentration up to 200 μg/mL. Cellular proliferation and induction of apoptosis or necrosis were monitored during a period of 24–72 h, showing that none of the powders tested caused cytotoxic effects. CNPs did not impair cellular proliferation, as shown by cell counts (Fig. 6a), neither they induced a significant increment of basal apoptosis or necrosis. Moreover, CNPs reduced basal ROS levels (significantly for C2, C3 and C4), as shown in Fig. 6b.

### CNP biological activity on Jurkat cells

CNPs are known to protect cells from oxidative stress sources (e.g. H_2_O_2_, UV rays, ionizing radiation) buffering ROS and therefore reducing oxidative stress damage to cellular bio-macromolecular structures^12, 29, 50, 69, 72^. This protective action against a superimposed oxidative stress condition was tested with the aim to compare the biological activity of the different CNP samples. To this purpose, Jurkat cells were pre-incubated with CNP suspensions at 100 μg/mL, and then treated with a strong oxidant agent, hydrogen peroxide (H_2_O_2_).

**Figure 7:**
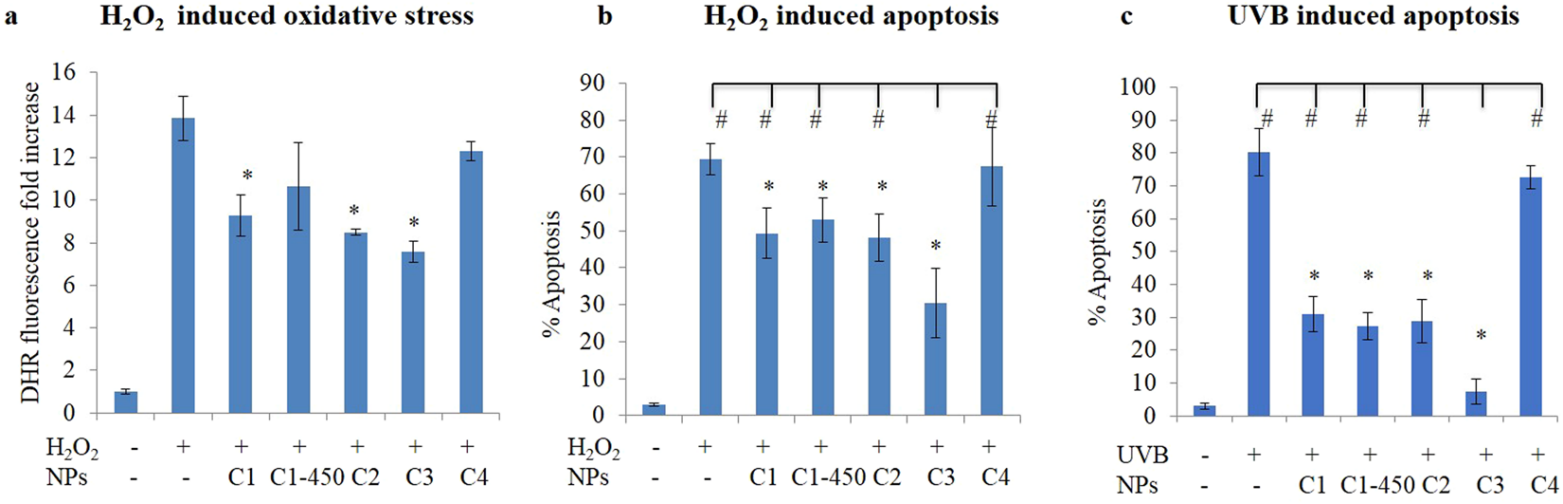
Redox active CNPs protect cells from hydrogen peroxide and UVB. (**a**) ROS levels increment measured by DHR fluorescent signal detected by flow cytometry 1 h after cell treatment with H_2_O_2_ ± CNPs. (**b**) % of apoptosis detected 24 h after cell treatment with H_2_O_2_ ± CNPs. (**c**) % of apoptosis detected 24 h after cell treatment with UVB ± CNPs. Values are the mean of ≥3 independent experiments ± SD; *p < 0.05 (ANOVA). Significance with respect to the control group is shown. In (**b**) and (**c**) also the significance with respect to the C3 group is shown, ^#^p < 0.05 (ANOVA).

Figure 7 shows the CNP protection against H_2_O_2_ and UVB induced ROS production and apoptosis. All the CNPs tested, except for C4, significantly reduced the extent of hydrogen peroxide noxious action, buffering the increase in ROS levels (Fig. 7a) and protecting cells from apoptosis (Fig. 7b). C3 sample was significantly more effective than the other samples, as confirmed by ANOVA mean comparison analysis between the different treatments (^#^p < 0.05). Figure 7c shows that the functionalized particles maintain a strong protecting activity against UVB-induced apoptosis^50^; in particular, C3 exerts an anti-apoptotic effect significantly stronger that the pristine particles.

To further compare the extent of antioxidant activity of the different CNP samples and their ability to inhibit the redox-dependent apoptotic process, we followed the approach reported by Celardo *et al*.^36^. Apoptosis was induced by treating cells with VP16, a topoisomerase II inhibitor. As reported in Supplementary Fig. S7, pre-treatment with the C1, C2 and C3 samples at a final concentration of 100 μg/mL significantly reduced VP16 induced apoptosis. Only the C4 sample was completely ineffective. Confirming the results obtained with H_2_O_2_ treatment, C3 showed a significantly higher inhibition of apoptosis than the other powders, suggesting that the biological activity of the different CNPs powders strongly correlate with the CNP water dispersibility.

**Figure 8:**
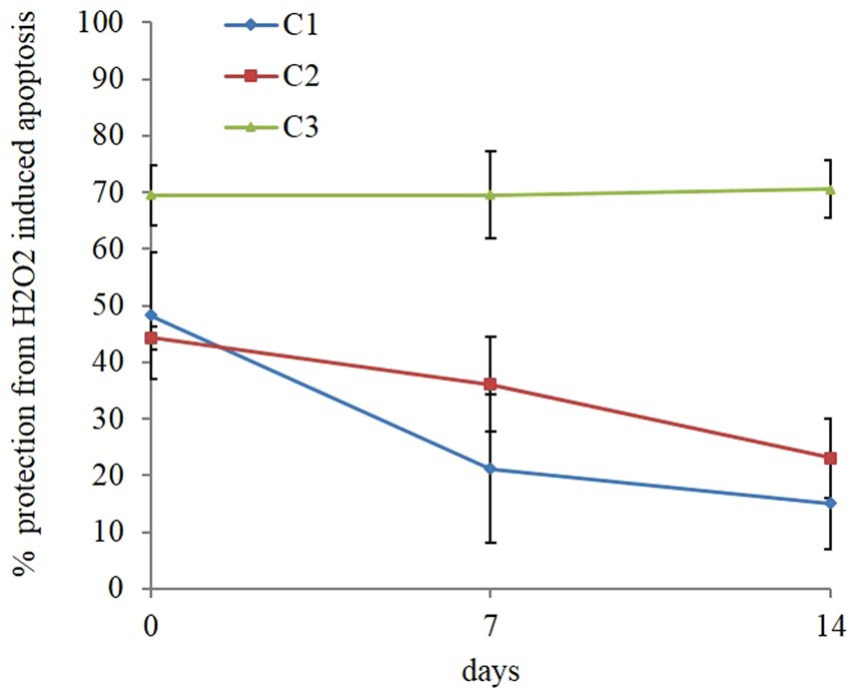
CNP redox activity over time. % protection from apoptosis in cells treated with H_2_O_2_ ± C1, C2, and C3 supernatant suspensions after 7 and 14 days. Values are the mean of ≥3 independent experiments ± SD.

Finally, we investigated whether the CNP supernatants maintained their biological activity over time. Therefore, CNP stock solutions were let to sediment at ambient conditions for 7 and 14 days and their activity tested as the protection from H_2_O_2_-induced apoptosis; cells were pre-incubated with the supernatant for 1 h (same volume of stock solution as in the previous experiments) and treated with H_2_O_2_. CNP supernatant ability to protect from H_2_O_2_-induced apoptosis is reported in Fig. 8. The supernatant recovered from C3 (which contained about 74 wt% of the pristine NPs) maintained its full activity for two weeks. On the other hand, C1 and C2 supernatant activity progressively reduced, even though not completely abolished, after 14 days.

In summary, different approaches to synthesize new biocompatible CNPs for medical applications were investigated. The previously reported TEMED induced precipitation method in the presence of Pluronic F127 was improved with the result of reducing CNP agglomeration tendency and enhancing their biological activity. An ethylene glycol assisted precipitation method followed by an *in situ* silanization with a poly(ethylene glycol)-terminated silane (MEEETES) was developed, significantly improving the CNP water-dispersibility. The nanoparticles obtained were highly stable in water for weeks. Moreover, their surface coating did not induce any toxicity neither influenced their redox activity. Importantly, all the particles maintained satisfactory redox activity: in particular, MEEETES functionalized nanoparticles showed a significantly higher biological activity (*vs* non-functionalized particles) that strongly correlates with their higher stability in physiological media, pointing to its use in further biological antioxidant applications. In conclusion, we showed that the water/ethylene glycol-based precipitation synthesis followed by *in situ* surface functionalization with a poly(ethylene glycol)-terminated silane is a promising method to obtain CNPs potentially highly active in biomedical applications.

## Methods

### Materials

All the reagents and solvents were purchased from Sigma-Aldrich, with the exception of 6-{2-[2-(2-Methoxy-ethoxy)-ethoxy]-ethoxy}-hexyl)triethoxysilane (Sikemia) and Dihydrorhodamine (Molecular Probes). Stock solutions of Hoechst 33342 (10 mg/mL), and PI (5 mg/mL) were dissolved in distilled water; DHR (10 mM) was dissolved in dimethyl sulfoxide.

### CNP synthesis

#### Method 1

Pluronic F-127 (6.5 g, 0.08 mol) was dissolved in 300 mL of Milli-Q water. After 1 h, Ce(NO_3_)_3_ × 6H_2_O (15.49 g, 0.036 mol) was poured into the solution, followed by the addition of N,N,N0,N0-tetramethylethylenediamine (TEMED 17 mL, 0.113 mol). The solution was kept overnight under mild stirring. NPs were washed by centrifugation in H_2_O and then dried overnight at 80 °C. The obtained material was ground in an agate mortar. To obtain C1–450 °C samples, the powders were annealed at 450 °C for 8 h.

#### Method 2

Ethylene glycol 95% (7.8 mL, 0.12 mol) was dissolved in 92 mL of Milli-Q water. Then Ce(NO_3_)_3_ × 6H_2_O (5.16 g, 0.012 mol) was poured into the solution, followed by the addition of NH_3_ (4.5 mL) to reach pH 9.6. The solution was kept at 50 °C under mild stirring until it became yellow. 50 mL out of 100 mL were then collected. The NPs (C2) were washed by centrifugation in EtOH and H_μ_O and dried overnight under vacuum at room temperature. 6-{2-[2-(2-Methoxy-ethoxy)-ethoxy]-ethoxy}-hexyl)triethoxysilane (MEEETES) (20 mM) was added to the residual solution (50 mL) and was kept overnight at 50 °C. The functionalized NPs (C3) were then washed by centrifugation in EtOH and H_2_O and dried overnight under vacuum at room temperature. 3-Aminopropyltriethoxysilane (APTES) functionalization (C4 sample) was obtained by a post synthesis functionalization of the dried C2 nanoparticles. The NPs (0.5 g) were dispersed in 50 mL of Milli-Q water. Then APTES (200 μL, 20 mM) was poured into the dispersion. The pH was adjusted to 9.6, then the reaction was kept overnight at 50 °C. The NPs were washed by centrifugation in EtOH and H_2_O and dried overnight under vacuum.

### Ce^3+^ coordination study

pH dependence of Ce^3+^ ions coordination by EG was studied by ^1^H-NMR titration performed in a Bruker 400 MHz. A solution of 1 mM Ce^3+^ and 1 mM EG (molar ratio 1:1) was prepared in D_2_O. NMR spectra were measured at different pH from 3 to 12, progressively adding NaOH.

### CNP crystal phase, size and morphology

Nanoparticle crystal phase was investigated by X-ray powder diffraction (XRD) analysis using a Philips X’Pert X-ray diffractometer. Crystal structure was identified by comparison of d-spacing values with the references taken from the JCPDS database (75–0390 for ceria fluorite structure). The crystallite size of samples, *dXRD* was estimated from XRD patterns by applying full-width half-maximum (FWHM) of (111) characteristic peak to Scherrer equation:

1 $$d{\rm{XRD}}=0.9\lambda /{\rm{FWHM}}\ast \,\cos \,\theta $$

where *λ* is the X-ray wavelength (1.5406 Å in this study) and *θ* is the diffraction angle for the (111) plane. Nanoparticle size and morphology were determined by transmission electron microscopy (TEM) observations, using a Philips/FEI CM12 operating at 100 kV.

### Nanoparticle ligand shell characterization

The amount of the CNP ligand shell was quantified by thermogravimetric analysis (TGA), using a Perkin Elmer TGA 4000, performed under nitrogen flow (20 mL/min) from 30 to 800 °C, with a 5 °C/min heating rate. The chemical composition of NP ligand shell was determined by ^1^H-NMR experiments, using a Bruker 400 MHz, and by Fourier-transform infrared spectroscopy (FTIR), using a Thermo Scientific Nicolet 6700 equipped with Smart iTR ATR.

### Dynamic light scattering and zeta potential measurements

A stock dispersion of CNPs was prepared in deionized water at the concentration of 20 mg/mL. Before performing the experiments, C1, C1–450, C2 and C4 samples were dispersed with ultrasounds (Branson Ultrasonic Corp., Danbury, CT, USA) at 20% amplitude for 3 minutes, and immediately diluted to the final concentration. C3 stock solution was only shaken for 30 seconds with a vortex and then diluted to the final concentration. As described also in ref. 50, zeta potential and dynamic light scattering (DLS) measurements of the NPs at the concentration of 200 μg/mL were performed at 37 °C in deionized H_2_O (pH 7.4) and in RPMI medium +10% fetal calf serum using a Malvern Zetasizer, Nano-ZS. DLS experiments consisted of 15 runs per measurement and all experiments were repeated three times. The mean ± standard deviation (SD) of the hydrodynamic diameter and the polydispersity index (P.I.) were calculated by cumulant analysis. Zeta potential experiments consisted of 100 runs per measurement and all experiments were performed three times. The mean of each triplicate measurement ± standard deviation (SD) was taken. Measurements in H_2_O were made in general purpose mode; measurements in cells media were collected in monomodal mode due to the high conductivity of this sample (16 mS/cm). The Smoluchowski approximation was used to convert the electrophoretic mobility to zeta potential. To check NP stability in water (pH 7.4) over time, CNP stock dispersions were let sediment at ambient condition for 2 weeks. Then, residual concentration into the supernatant was determined by measuring CNP suspensions UV-Vis absorption in the range of 200–800 nm using a UV-Vis spectrometer (Varian Cary 50).

### Electron spin resonance (ESR) spectroscopy measurements

CNP ability to scavenge hydroxyl radical was assessed by ESR. The ESR measurements were carried out at room temperature using a Bruker X-band ESR spectrometer, Bruker Spectrospin Model EleXsys 500, which was equipped with a super-high-Q cavity, Bruker Model ER 4122SHQE. HO^●^ radicals were generated by exposing aqueous suspensions of TiO_2_ (anatase) NPs, prepared as in ref. 73, to UV-A light (***λ***_**ex**_ = 365 nm), using a UV spot light source, Lightingcure™, model LC-8 (Hamamatsu Photonics, France). During exposure to light, the 2-mL volumes of suspensions were equilibrated with oxygen at the atmospheric pressure and stirred vigorously to prevent agglomeration of nanoparticles. To avoid overheating by light, the suspensions were maintained at a stabilized temperature of 25.0 ± 0.1 °C, using a Thermo Fisher Scientific Haake K10 bath vessel with a temperature control module. Two different experiments were performed to monitor the changes in hydroxyl radical concentrations in the presence of CNPs:

(i) 5,5-dimethyl-1-pyrroline N-oxide (DMPO) was used as a spin trap of HO^●^ radicals, thus leading to the formation of ESR detectable DMPO/OH spin adducts. Prior to ESR measurements, an aqueous dispersion of 800 μg/mL TiO_2_ ± 400 μg/mL CNPs was sonicated as previously described and then mixed with the stock solution of the spin trap to achieve the final DMPO concentration of 50 mM. Thus, the prepared solutions were exposed to UV-A light for determined time intervals, while being continuously stirred using a magnetic bar. After each illumination step, the suspensions volumes of *~*15 µL were drawn into thin-walled borosilicate glass capillaries (0.7 mm ID, 0.87 mm ID, Model CV7087-100, VitroCom Inc., Mountain Lakes, NJ, USA), sealed on both ends with a tube sealant, ChaSeal (Chase Scientific Glass Inc., Rockwood, TN, USA). Control ESR measurements were always performed for all the nanoparticle suspensions prior to exposing them to light. The typical instrumental settings during these measurements were: microwave frequency 9.399 GHz, microwave power 0.64 mW, sweep width 120 G, modulation frequency 100 kHz, modulation amplitude 0.5 G, receiver gain 60 dB, time constant 41 ms, conversion time 82 ms, and total scan time ~168 s. Routinely, to improve the signal-to-noise ratio, two traces were accumulated for each ESR spectrum. Control ESR measurements were performed for all the DMPO-nanoparticle suspensions prior to exposing them to light.
(ii) The ESR measurements of the light-induced decomposition of 4-hydroxy-2,2,6,6-tetramethylpiperidine 1-oxyl (TEMPOL), accompanied by the formation of 2,2,6,6-tetramethylpiperidinone 1-oxyl (TEMPONE), were performed by irradiating with UV-A light the aqueous solution of 100 μM TEMPOL containing also the dispersed nanoparticles of TiO_2_ and CeO_2_ (800 μg/mL TiO_2_ ± 400 μg/mL CNPs) for 30–525 s by implementing the same experimental conditions as described above. The typical instrumental settings while monitoring the light-induced decomposition of TEMPOL were: 9.399 GHz, microwave power 0.64 mW, sweep width 60 G, modulation frequency 100 kHz, modulation amplitude 0.5 G, receiver gain 60 dB, time constant 20.5 ms, conversion time 84 ms, and total scan time 84 s. The individual contributions of ESR signals of TEMPOL and TEMPONE to the overall ESR signal were quantified by simulating the respective ESR spectra with the help of WIN-ESR SimFonia.

### Cell cultures

Jurkat cells, human tumor T lymphocytes, were grown at 37 °C in RPMI 1640 medium supplemented with 10% fetal calf serum (FCS), 2 mg/mL L-glutamine, 100 IU/mL penicillin and streptomycin, in a humidified atmosphere of 5% CO_2_ in air. All experiments were performed on cells in the logarithmic phase of growth under condition of >96% viability. In each experiment, cells were kept at the concentration of 10^6^ cells/mL^36^.

### Cell treatment with CNPs

NP suspensions were prepared at a stock concentration of 20 mg/mL, dispersed as previously described and immediately incubated with Jurkat cells at the final concentration. To check the supernatant residual activity, CNP stock solutions were let to sediment for 1–2 weeks at ambient temperature. Then, parallel experiments were performed treating cells with the same volume of supernatant or of a freshly prepared stock solution.

### Analysis of cell proliferation

The rate of cell proliferation was assessed after incubating cells with CNPs up to 200 μg/mL concentration for 24–72 h, by evaluating cell concentration using a Bruker counting chamber; values are given as number of 10^4^ cell/mL.

### Detection and quantification of intracellular ROS

Reactive oxygen species (ROS) were measured by dihydrorhodamine (DHR), a probe that is internalized in cells and fluoresces only when oxidized to rhodamine^74^, allowing quantitative assessment of intracellular ROS. DHR was added directly to the cell samples to a final concentration of 2 µM and incubated at 37 °C in the dark for 20 min; then cells were analyzed by FACSCalibur flow cytometer; 10,000 cells were analyzed for each sample. Data were analyzed with WinMdi 2.9 software; the mean values were used for tables and graphs^50^.

### Induction and evaluation of apoptosis

Apoptosis was induced by the following agents:

(i) 200 μM hydrogen peroxide, added to medium culture;
(ii) 100 μM etoposide, a topoisomerase II inhibitor inducing apoptosis via DNA damage.
(iii) UVB (312 nm, Spectroline lamp model ENB-260C/FE) single exposure at 3 mW/cm^2^ for 1:30 minute.

Cells were pre-treated with 100 μg/mL CNPs 60 min prior to the experiment.

Apoptosis was evaluated quantifying the fraction of apoptotic nuclei by fluorescence microscopy after DNA staining with the cell-permeable specific dye Hoechst 33342, directly added to the cell culture at the final concentration of 10 µg/mL^36^. To evaluate the eventual presence of necrotic cells, cells were also stained with PI at a final concentration of 5 µg/mL. The fraction of apoptotic nuclei among the total cell population was calculated by counting at the fluorescence microscope at least 300 cells in at least three independent randomly selected microscopic fields.

### Statistical analysis

Each experiment was repeated ≥3 times. Data are presented as means ± SD. Statistical evaluation was conducted by one-way ANOVA analysis, followed by Tukey’s Multiple Comparison Test (Homogeneous Variances) using the software Origin 8.0. Statistical significance was set at p < 0.05.

## Electronic supplementary material

Supplementary Information
