# Supplementary material for: A novel synthetic approach of cerium oxide nanoparticles with improved biomedical activity
Source: Sci Rep. 2017 Jul 5;7:4636. doi: 10.1038/s41598-017-04098-6 (PMC5498533; doi:10.1038/s41598-017-04098-6)
Supplement: Supplementary file 1 — Supplementary Information [file 41598_2017_4098_MOESM1_ESM.pdf]

Supplementary Information

A novel synthetic approach of cerium oxide nanoparticles with improved biomedical activity

Fanny Caputo,[1,2] Marta Mameli,[3] Andrzej Sienkiewicz,[4] Silvia Licoccia,[1] Francesco Stellacci,[3] Lina Ghibelli,[2] Enrico Traversa[1,5,*]

[1] Dipartimento di Scienze e Tecnologie Chimiche, Università di Roma Tor Vergata, 00133 Roma, Italy
[2] Dipartimento di Biologia, Università di Roma Tor Vergata, 00133 Roma, Italy
[3] Institute of Materials, Ecole Polytechnique Fédérale de Lausanne, 1015 Lausanne, Switzerland
[4] Institute of Physics, École Polytechnique Fédérale de Lausanne, 1015 Lausanne, Switzerland
[5] International Research Center for Renewable Energy, Xi'an Jiaotong University, 710049 Xi'an, Shaanxi, China
* traversa@uniroma2.it

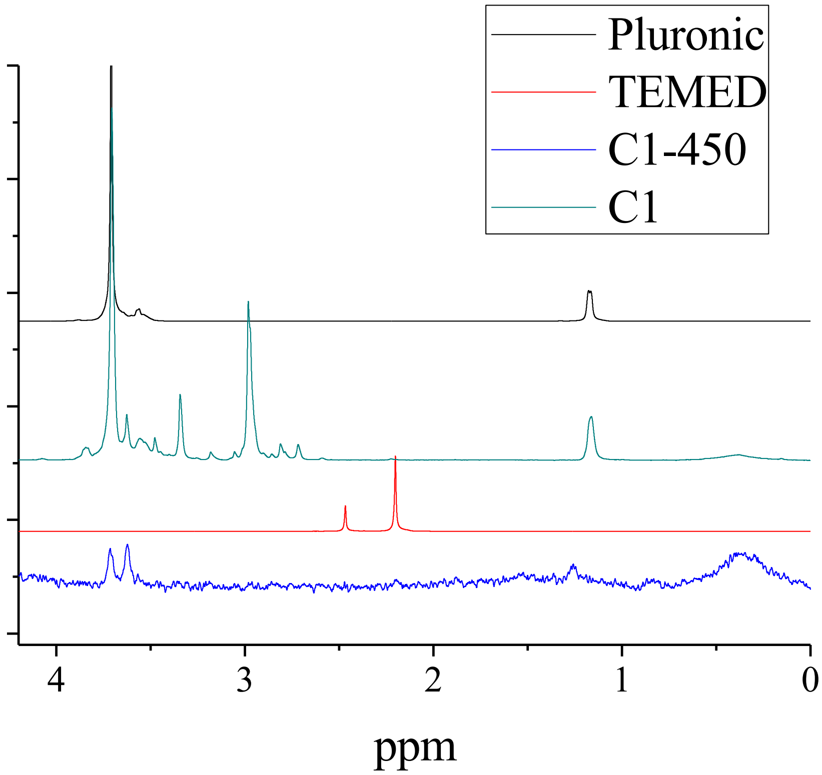

Figure S1. ^{1}H-NMR surface characterization of C1. *C1 ^{1}H-NMR spectrum compared with Pluronic F127, TEMED spectra and C1-450 NPs.*

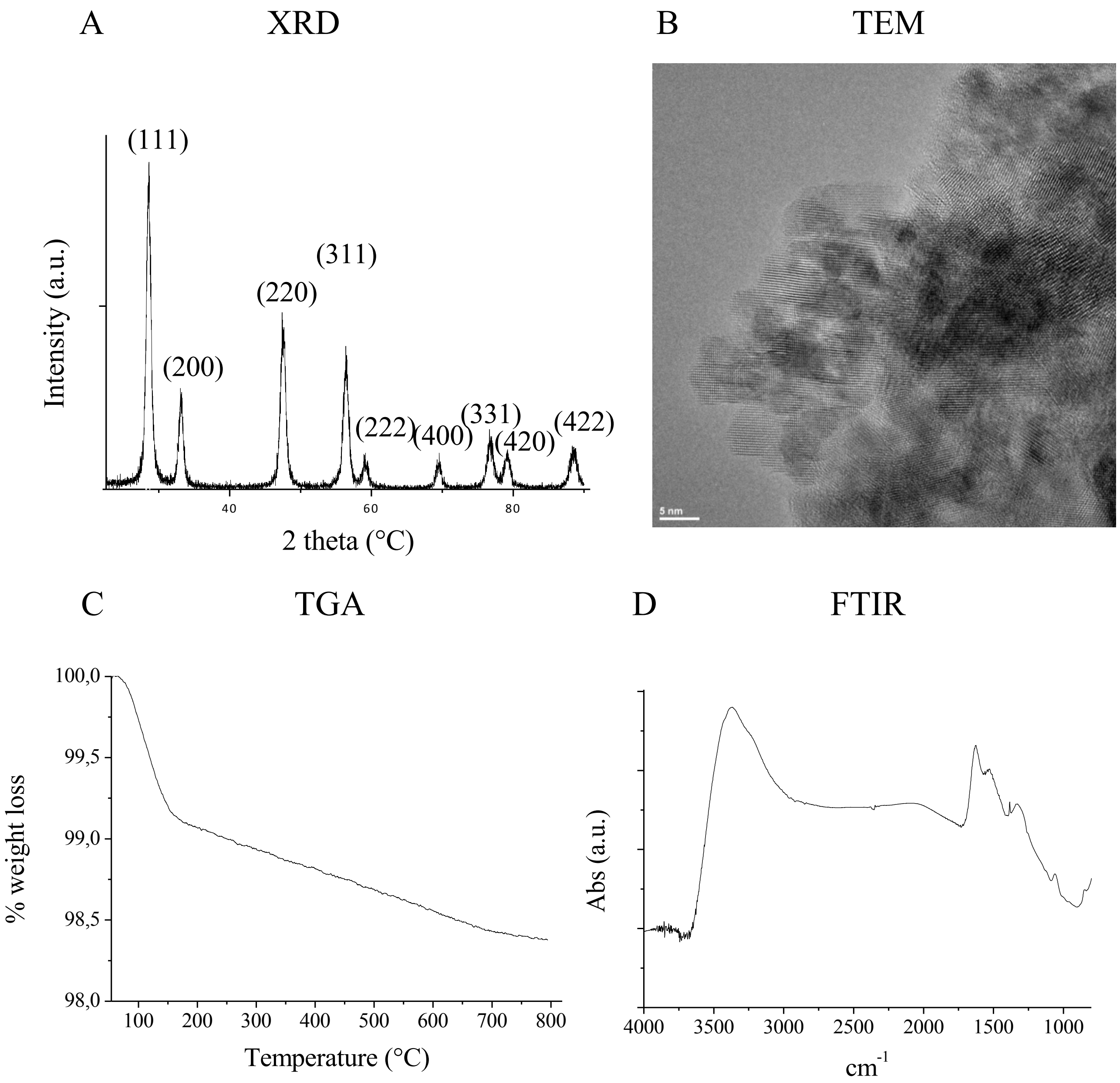

Figure S2. C1-450 Physico-chemical characterization. *(A) XRD diffraction patterns and Miller indexes. (B) TEM image. (C) TGA curve. (D) FTIR absorbance spectra.*

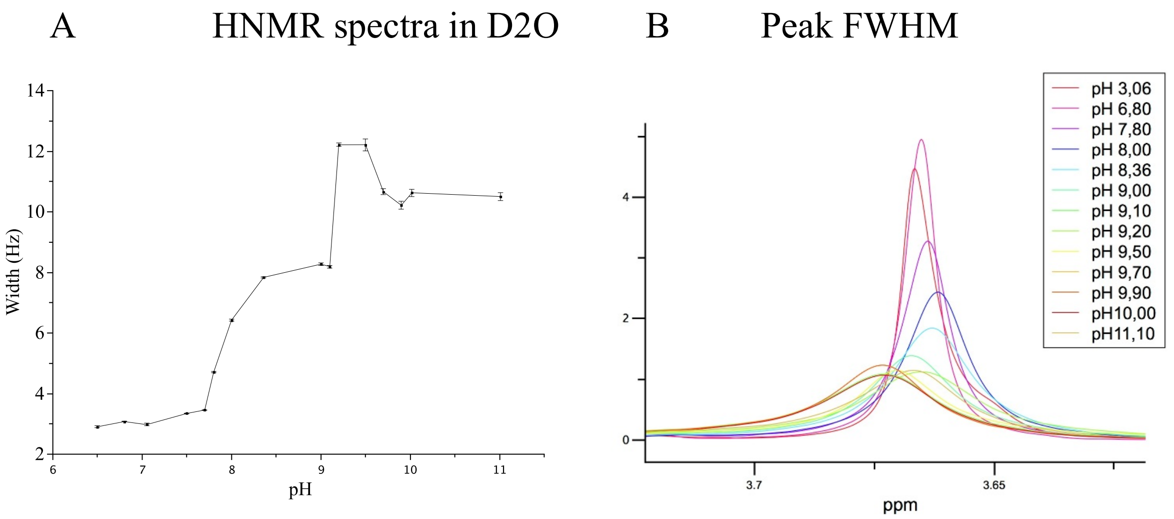

Figure S3. Ce (III)-EG coordination depends on pH. *(A) ^{1}H-NMR spectra of EG peak at 3.66 ppm varying the pH from 3 to 11. (B) Dependence of peak full width at half maximum (FWHM) on the pH.*

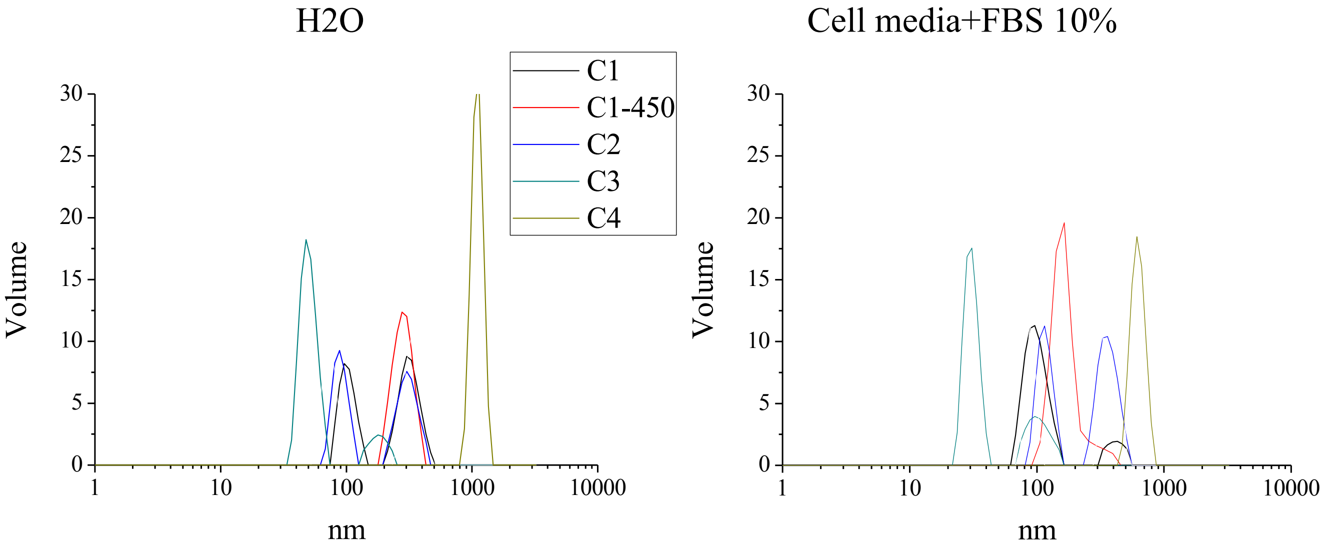

Figure S4. CNP dispersion properties. *DLS analysis of C1, C1-450, C2, C3, and C4 at 200 µg/mL in water (pH 7.4) and in RPMI media +10% fetal bovine serum (FBS). The hydrodynamic diameter distribution by volume is reported.*

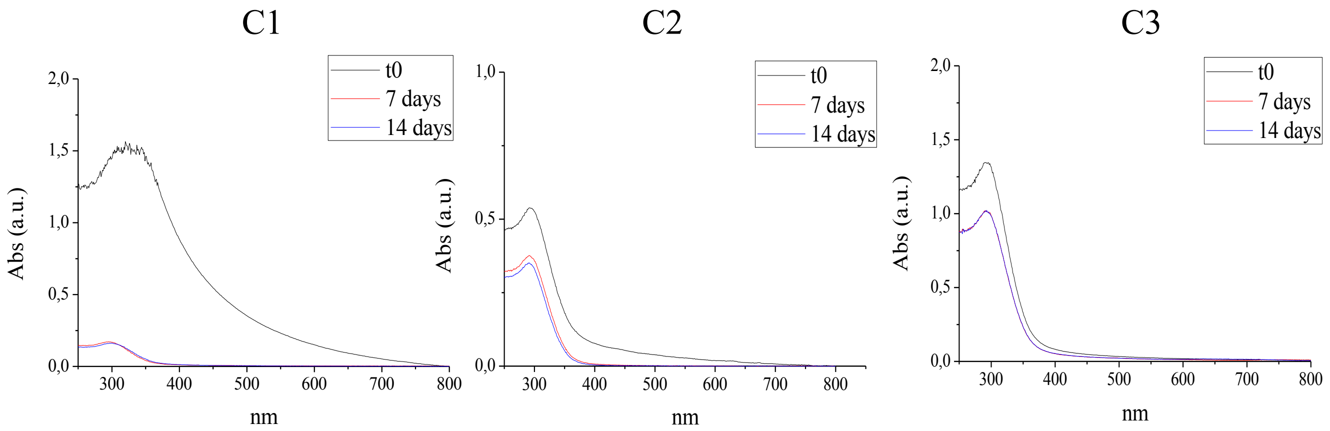

Figure S5. CNP precipitation analysis over time. *Time dependent variation of CNPs UV-VIS supernatant spectra after 7 days (red curve) and 14 days (blue curve) compared with CNP spectra immediately after sonication (black curve). CNP nominal concentration was set at 50 µg/mL.*

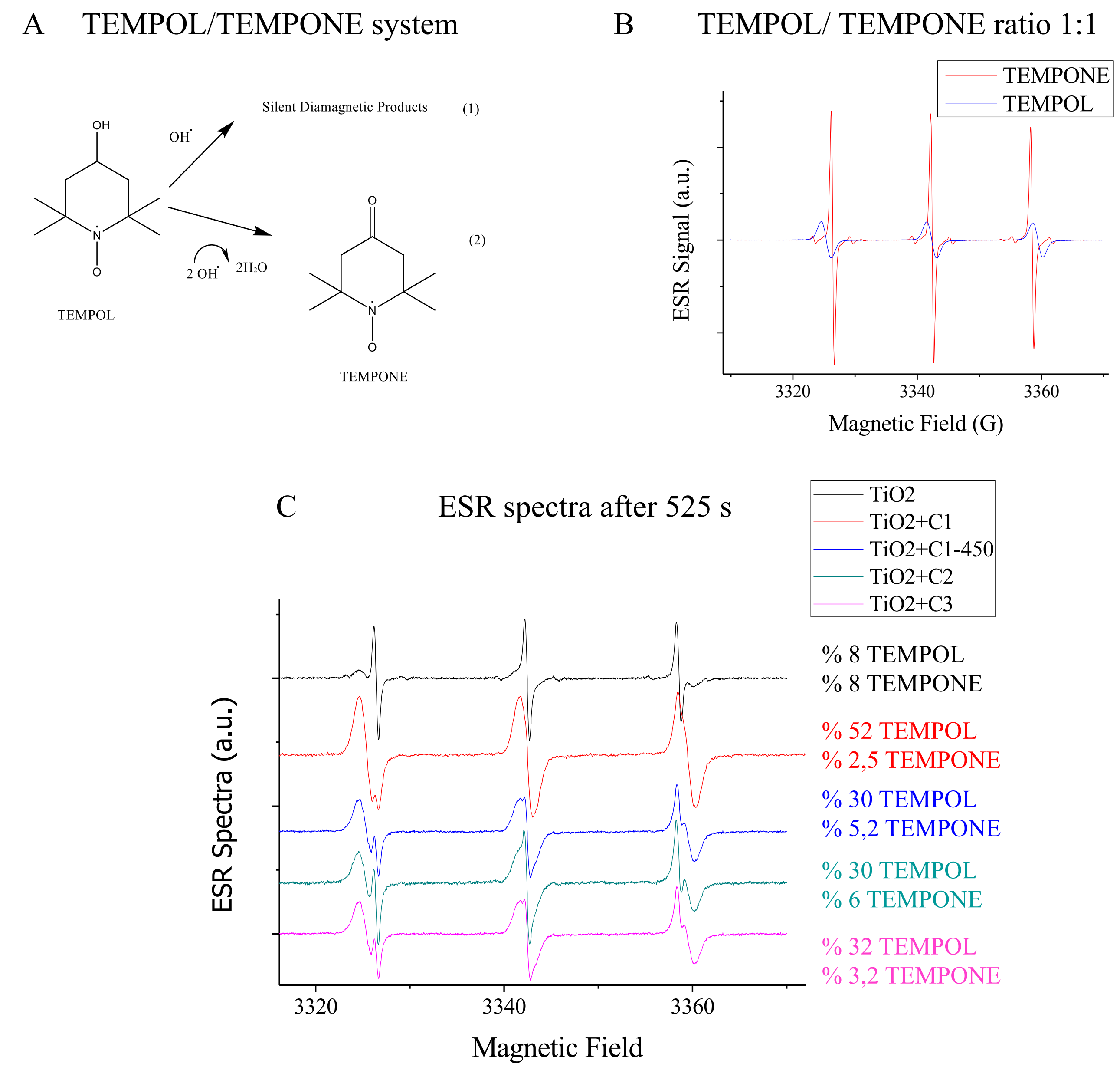

Figure S6. TEMPOL/TEMPONE ESR system. *(A) Scheme of TEMPOL degradation by* $HO^{\bullet}$ *producing diamagnetic products (reaction 1), and paramagnetic TEMPONE species (reaction 2). (B) Simulation of TEMPOL (blue curve) and TEMPONE (red curve) ESR spectra (TEMPOL: TEMPONE ratio 1:1). TEMPOL spectra is shifted to the right by 0.035 G. (C) ESR spectra of 100 μM TEMPOL after 525 s of continuous production of* $HO^{\bullet}$ *± (C1, C1-450, C2 and C3 samples) as described in Methods section. For each spectra the quantification of TEMPOL and TEMPONE signals are reported in the bottom right.*

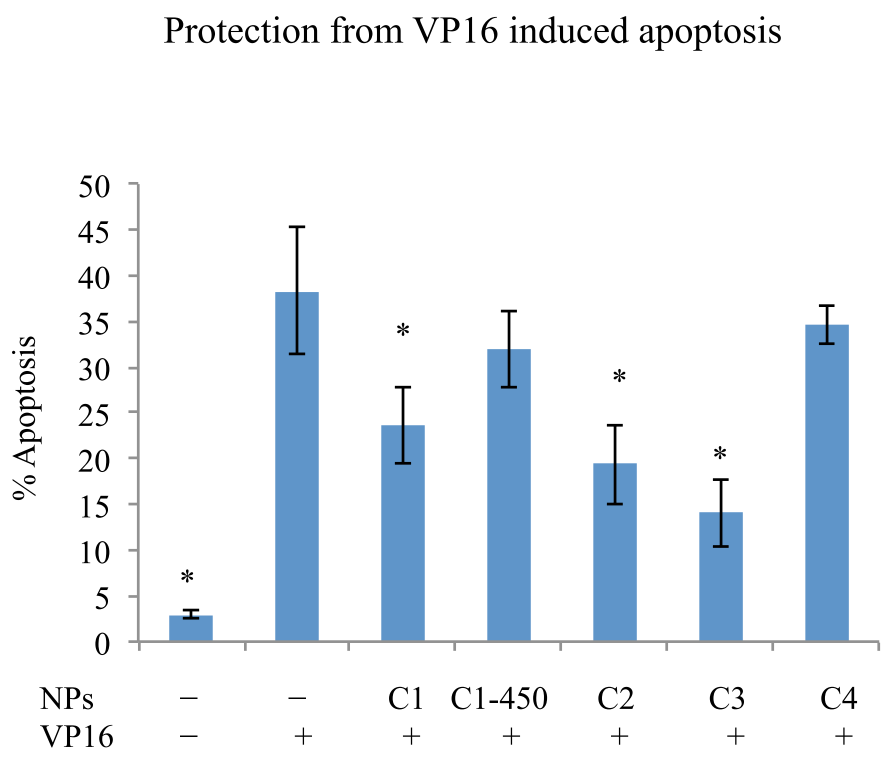

Figure S7. Redox active CNPs reduce redox dependent apoptosis*. % of apoptosis detected 3 h after cell treatment with VP16 ± CNPs. Values are the mean of ≥ 3 independent experiments ± SD; *$p < 0.05$ (ANOVA). Significance with respect to the control group is shown.*
